# Emerging Nanocarrier Strategies for Optimized Drug Delivery in the Treatment of Primary Bone Cancer

**DOI:** 10.32604/or.2026.077723

**Published:** 2026-06-16

**Authors:** Lindokuhle M. Ngema, Samson A. Adeyemi, Yahya E. Choonara

**Affiliations:** 1 Wits Advanced Drug Delivery Platform Research Unit, Department of Pharmacy and Pharmacology, School of Therapeutic Sciences, Faculty of Health Sciences, University of the Witwatersrand, 7 York Road, Parktown, Johannesburg, South Africa; 2 Department of Biomedical and Nutritional Sciences, Zuckerberg College of Health Sciences, University of Massachusetts Lowell, Lowell, MA, USA; 3 Infectious Diseases and Oncology Research Institute (IDORI), Faculty of Health Sciences, University of the Witwatersrand, Parktown, Johannesburg, South Africa

**Keywords:** Bone cancer, drug delivery, bone microenvironment, bone marrow, nanocarriers, nanomedicine

## Abstract

Primary bone cancer is a relatively rare malignant tumor that manifests in the bone and affects the normal functioning of the bone tissue. Primary bone cancer can be characterized into three subtypes, which are osteosarcoma, chondrosarcoma, and Ewing sarcoma. Notably, the treatment of primary bone cancer with conventional modalities, like chemotherapy and surgical interventions, has been overwhelmed with dismal clinical outcomes. The conventional therapies are challenged with non-specificity, resulting in off-target effects and ultimate harm to healthy tissue. Particularly, chemotherapy as a first-line treatment option is riddled with poor drug bioavailability, limited tumor accumulation, and increasing drug resistance. Several innovative drug delivery systems, including nano-based carriers, have been investigated to overcome the systemic drug delivery challenges in primary bone cancer. Accordingly, with most reviews focusing on bone metastasis (secondary bone cancer), this current narrative review aims to provides critical insights on nanocarrier strategies for drug delivery in primary bone cancer, comprehensively expounding on the epidemiology, cellular mechanisms, and etiological effects of primary bone cancer, as well as the current therapies and new drug nanocarriers prototyped to optimize the clinical outcomes in bone cancer management.

## 1 Introduction

Bone cancer is amongst the least common types of malignancies, making up less than 1% of reported cancer cases globally [1]. Primary bone cancer is predominant in young adults and children, and can be categorized into three subtypes, namely osteosarcoma, chondrosarcoma, and Ewing sarcoma [2]. These develop from the different parts of the bone [3]. Ewing sarcoma is the least prevalent of the subtypes often originating from the tissue surrounding the bone, and constitutes about 16% of diagnosed primary bone cancer cases, followed by chondrosarcoma (~20%), which develops from the cartilage cells, and at the top is osteosarcoma (~35%), which develops in the growing ends of the bone [2]. Besides primary bone cancer, another form of bone malignancy, referred to as bone metastases, is prevalent in patients already presenting with another type of cancer in the body [4]. Essentially, bone metastases originate from other cancer sites and spread to the bones. The current review is, however, solely focused on primary bone cancer, which originally manifests in the bone, from the rampant bone cells.

Primary bone cancer accounts for approximately 0.2% of all confirmed cancer mortalities and nearly 5% cancer-related deaths in children worldwide [2]. About 3600 new cases were estimated in 2020 in the United States, with about 1720 reported deaths. The global osteosarcoma female: male ratio is reportedly 1:1.43, which demonstrates the predominance of primary bone cancer in males [5]. Essentially, osteosarcoma has been shown to account for the majority of incidences in children and the elderly. Particularly, African countries like Nigeria, Uganda, and Sudan are amongst the regions with the highest recorded cases of osteosarcoma [6]. The American Cancer Society had reported that ~2160 incidences were recorded in males and ~1810 in females in 2023, driving the total of new cases to ~3970 [7]. Likewise, the number of deaths in males is estimated at 1200, meanwhile 940 deaths are expected in females, totalling about 2140 deaths in 2023 [7].

The ultimate risk factors of primary bone cancer remain poorly understood. However, it has been deduced that socio-demographics (i.e., gender and age), radiation exposure, and genetics may play a key role in the manifestation of primary bone cancer, particularly in children [8]. Hereditary factors have been implicated in the etiology of bone cancer from earlier investigations, with studies involving children and meta-analysis investigations in which cases of osteosarcoma development are documented [9]. Meanwhile, gender and race are also reported to contribute as plausible factors in the high incidences of primary bone cancer [10]. Additionally, unchecked exposure to radiation, particularly at an early age, increases the risk of bone cancer, in which, in most cases, the cancer may manifest in 5–20 years post exposure [8].

Sadly, the therapeutic approach for primary bone tumors has been largely focused on attaining local tumor control while sustaining the patients’ quality of life [11]. Currently, chemotherapy (i.e., neoadjuvant and/or adjuvant), radiotherapy, and surgery are the best treatment options for bone cancer [12]. Accordingly, several chemotherapeutic regimens have been passed by the U.S Food and Drug Administration (FDA) for use in the treatment of bone cancer, whether individually or in combination. Amongst these chemotherapeutic drugs are methotrexate, doxorubicin, ifosfamide, and cisplatin [12,13]. However, the use of chemotherapy regimen in bone cancer comes with significant challenges, such as non-specific distribution, poor accumulation into tumors, rapid clearance, and high toxicity, resulting in unwanted side effects [12].

Furthermore, the efficient delivery of therapeutics to the bone tissue remains a major challenge, due to the complex anatomical and physiological attributes of bone tissue [14]. Generally, drugs can be delivered to the bone tissue either locally (direct injection to bone tissue, i.e., use of drug eluting implants) or systemically (via bloodstream, i.e., using drug carriers) [15,16]. The structural composition of the bone is primitively maintained through the bone remodelling process, which involves the replacement of the damaged or old bone by the osteoclasts, and replacement with newly formed bone by the osteoblasts [14,17]. Moreover, 80% of bone mass comprises a mixture of calcium phosphate salts and collagen, making bones exceptionally resistant to mechanical stress, while structurally strong enough to carry the whole body [18]. Although highly vascularized, the bone vascular network is structurally varied based on skeletal site, age, as well as existence of a disease condition, which may compromise the diffusion of molecules from the bloodstream to the target location [18]. Likewise, when the basal membrane is present in the blood vessels, drug penetration and accumulation in the bone may be hindered [14].

Consequently, different drug delivery systems have been proposed to overcome these hurdles, offering enhanced therapeutic effects to prolong the survival of patients and enhance the quality of life. These new drug delivery systems, including nanoparticle-based platforms, have demonstrated some advantages over conventional therapies for managing primary tumors, with minimal adverse effects and enhanced drug pharmacokinetics reported in pre-clinical and clinical studies.

The current review aims to discuss these drug delivery systems in detail and provides insights into key aspects of primary bone cancer, such as cellular mechanisms, etiology, and epidemiology, which inform the design of emerging nano-based therapies for primary bone cancer.

## 2 Classification of Primary Bone Cancer: Subtypes and Diagnosis

**Figure 1:**
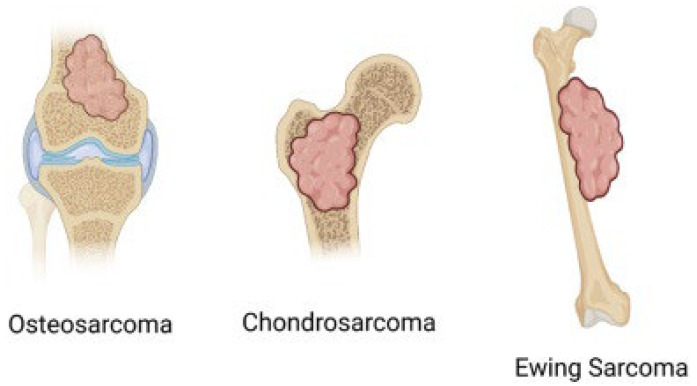
**Subtypes of primary bone cancer and their sites of origin.** Osteosarcoma originates in the growing ends of the bone, and chondrosarcoma develops from the cartilage cells in the bone, meanwhile Ewing sarcoma develops from the tissue surrounding the bone. Reproduced with permission from [3], under a Creative Commons CC-BY-NC-ND license. Copyright ©2023 Elsevier B.V.

Primary bone cancer categorically refers to tumors that specifically occur in bone as a site of origin, in contrast to secondary bone cancer, which refers to other cancers that metastasize to the bone tissue [19]. These tumors develop from the different parts of the bone, as shown in Fig. 1 [3], and are categorized into (i) osteosarcoma which mostly manifests in the growing ends of the arm and leg bones of young adults and children, (ii) chondrosarcoma which develops from the cartilage, and (iii) Ewing sarcoma which popularly originates from the surrounding tissue in the pelvis, arms, or legs [19]. Early diagnosis of bone cancer plays a key role in the appropriate staging and prognosis, as well as apt intervention for limiting morbidity and mortality [17,20].

The diagnosis of primary bone cancer is typically informed by the primary symptoms and indications, including abnormal serum levels of calcium and alkaline phosphatase, pathological fractures, and bone pain, along with complementary data from imaging evaluations [20]. The diagnosis is generally achieved through surgical based biopsies and imaging examinations like magnetic resonance imaging (MRI), computed tomography (CT), X-ray, and positron emission tomography (PET) [19]. In essence, the diagnosis of bone cancer needs to be accurate and timely, as the stage of the cancer influences the choice of intervention and ultimately the patients’ survival [17,20]. This section discusses the three main subtypes of primary bone cancer and the relevant diagnostic modalities used in their evaluation.

### 2.1 Osteosarcoma

Osteosarcoma, most common in adolescents and young adults, is the most prevalent subtype of primary bone tumors constituting almost 35% of all reported primary bone cancer cases [1,2]. The excessively aggressive tumor is mostly secondary in patients over 40 years, particularly males, as it arises post exposure to chemical agents, radiation, or viruses, and may be considered distinct from osteosarcoma in children based on histogenesis [21]. Osteosarcoma is marked by osteoid production and disorganized bone structure appearing as a fine lacey trabecular pattern, mostly manifesting at the metaphysis of rapidly growing long bones [12]. In addition, osteosarcoma is genetically characterized by complex cytogenetic changes, consisting of both structural and numerical changes, which often amplify and yield a hyperploid genotype [22].

The diagnosis of osteosarcoma is generally achieved through an interdisciplinary cooperation amongst oncologists, orthopaedic surgeons, pathologists, and radiologists [22]. Conventional osteosarcoma exhibits a highly variable radiographic appearance, as the tumor mostly presents as a blended lytic lesion with cortical degradation [23]. Nonetheless, the osteoid-extracellular matrix is the characteristic feature of osteosarcoma, and its nature determines the histological subtype, which subsequently predicts survival [22,23]. Accordingly, conventional radiography plays a key role in detecting primary tumors, whereas examination through magnetic resonance imaging (MRI) or dynamic enhanced MRI is employed in detecting tumor extension in adjacent joints, as well as its association with soft tissue components [22]. Additionally, dynamic enhanced MRI can be employed in monitoring the effects of neoadjuvant chemotherapy before surgery [22].

### 2.2 Chondrosarcoma

Chondrosarcomas are rare malignant cartilaginous neoplasms characterized by variously differentiated chondroid matrix producing cells [12,24]. Chondrosarcomas account for approximately 10–20% of all reported malignant bone tumors, and in contrast to osteosarcoma and Ewing sarcoma, these mesenchymal tumors are prevalent in adults, with more than 70% of diagnosed cases manifesting in people above the age of 40 [12]. Moreover, chondrosarcomas present with diverse morphological and clinical features, which categorize this heterogeneous group of tumors into primary/conventional chondrosarcoma (80–90%) and differentiated/secondary chondrosarcoma (10%) [12,24]. Conventional chondrosarcoma normally affects the pelvis, humerus femur, ilium, and ribs; however, it has a high tendency to spread to other organs, particularly lungs [12].

Generally, a definitive diagnosis of chondrosarcoma can be achieved from imaging examination only, owing to the characteristic radiographic features of the lesions [24]. As such, plain X-ray is employed to identify the cartilaginous nature of the lesion and often reveals intralesional calcifications, endosteal scalloping, and permeative appearance in high-grade chondrosarcomas [24,25]. Similarly, CT scan and MRI can be employed to reveal these characteristic features of chondrosarcomas, including matrix calcification in over 90% of the cases and a cortical breach in the examination of long bone neoplasms [24]. On the other hand, tumor biopsy remains essential for diagnosing chondrosarcoma and differentiating it from benign and other malignant bone tumors [25].

### 2.3 Ewing Sarcoma

Ewing sarcoma is a highly malignant form of sarcoma which originates mostly from the inner part of the bone, and predominantly manifests in adolescents and young adults [26,27]. Although uncommon (constituting ~16% of all diagnosed bone sarcomas), Ewing sarcoma remains the most undifferentiated group of a primitive neuroectodermal tumor with a high probability of recurrence [26]. The Ewing sarcoma family comprises classic Ewing sarcoma of bone, malignant small-cell tumor of the chest wall (Askin tumor), extra-skeletal Ewing sarcoma, as well as soft-tissue based primitive neuroectodermal tumors. These are characterized by systematic chromosomal translocations that yield fusion genes encoding aberrant transcription factors, and mostly affect anatomic sites like the pelvis, femur, and axial skeleton, although they may also manifest in almost any other tissue [26,27]. The apparent indication of Ewing sarcoma in most patients is the swelling and pain over the site where the tumor is located [28].

Appropriate diagnosis of Ewing sarcoma involves imaging, which is a decisive factor for both diagnostic and therapeutic evaluation [26]. Accordingly, the initial staging and follow-up protocol are informed by the imaging data. The primary diagnosis, particularly in children, is based on MRI, which visualizes the local magnitude of the tumor, with subsequent projection radiography and/or CT carried out if the MRI gives inconclusive findings [26,27,28]. Radiography and CT findings that are common in Ewing sarcoma and possible malignant bone neoplasms include permeative osteolysis and mineralization of matrix [26]. Meanwhile, the signs of a suspected Ewing sarcoma on MRI include bone marrow displacement and the extraosseous tumor extension, as well as joint infiltration [28].

## 3 Bone Biology and Cellular Mechanisms Implicated in Primary Bone Cancer

**Figure 2:**
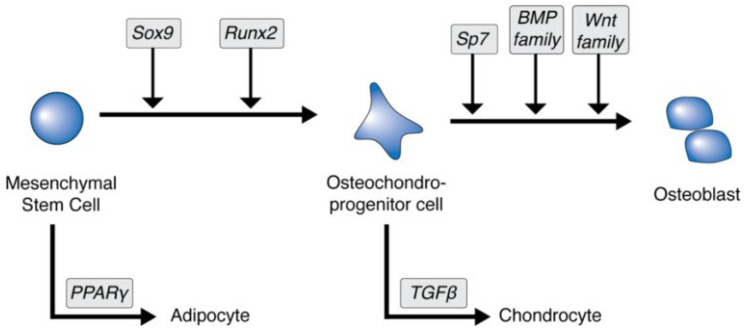
**Differentiation of Mesenchymal stem cells into different cell types is driven by the expression of certain protein families and transcription factors.** Peroxisome proliferator-activated receptor gamma (PPARγ) regulates the differentiation of mesenchymal stem cells into adipocytes, with sex determining region Y (SRY)-box transcription factor 9 (SOX9) and Runt-related transcription factor 2 (Runx2) driving differentiation into osteochondroprogenitor cells. Further differentiation into chondrocytes can be driven by the transforming growth factor-beta (TGFβ) expression, which can also stimulate calcium deposition and alkaline phosphatase (ALP) activity. Sp7: osterix; BMP: bone morphogenic protein; Wnt: wingless and int-1. Reproduced with permission from [22], under a Creative Commons Attribution (CC BY) license. Copyright ©2021 MDPI, Basel, Switzerland.

Bone cancer normally targets long bones (i.e., humerus, tibia, femur) close to the metaphyseal growth plate, presenting with high cell turnover and metabolic activity [11]. Essentially, the bone comprises four major cell types, namely osteoblasts, osteocytes, osteoclasts, and bone-lining cells. Meanwhile, the bone microenvironment consists of the cartilage surrounding bone, which is made up of bone marrow-derived mesenchymal and hematopoietic stem cells, as well as chondrocytes (endothelial cells and fibroblasts that make up the bone stroma) [22]. Drug delivery to the bone remains a significant challenge, owing to the unique anatomical and physiological characteristics of bone tissue [14]. Although the bones are highly vascularized, the structure of the vascular network can vary greatly with age, compromising the drug diffusion from the bloodstream to the target site [29]. Likewise, the presence of the basal membrane in some bone blood vessels may hinder drug penetration and accumulation at the target site [14,29]. These are key challenges that limit the therapeutic efficacy of current primary cancer therapies, thus necessitating the development of nanocarriers. The signaling factors of the bone microenvironment play a key role in the development of bone cancers. Mesenchymal stem cells are common precursors for chondrocytes, fibroblasts, as well as osteoblasts [22,30]. The differentiation of stem cells is in response to the absence or the expression of various transcription factors, as depicted in the example in Fig. 2 [22].

The manifestation of bone cancer primarily derives from transformed osteoblastic cells emanating from pre-osteoblasts experiencing Rb and p53 pathway aberrations, combined with certain genetic mutations [31]. These aggressively growing cells then disrupt the dynamics of bone remodelling, resulting in the eruption of hostile osteolytic lesions, which cause bone fragility. The rapid osteoclastic activity associated with abnormal receptor activator of nuclear factor-κB ligand (RANKL)/RANK pathway results in the discharge of growth factors from the bone matrix, such as transforming growth factor-β (TGF-β) and insulin-like growth factor-1 (IGF-1), which are involved in primary tumor growth [11,31]. Moreover, growth-related factors may be implicated in bone tumorigenesis as they are pivotal for the osteoblast-regulated bone formation [31].

Factors present throughout the bone microenvironment also play a role in the abnormal activity of osteoclasts, which can subsequently result in bone tumor proliferation [31]. As osteoclasts are driven by RANK signalling, which is modulated by osteoblasts’ RANKL expression, the disruption in RANKL expression and ligand binding by osteoblasts can thus halt bone resorption by osteoclasts and promote unchecked bone formation [22]. Likewise, factors released by cancer cells, such as interleukins (IL), i.e., IL-11 and IL-6, and TGF-β, can also facilitate RANK expression on the surface of osteoclasts, which can enhance bone resorption and promote tumor progression [22,31]. The bone microenvironment also allows many other cancers to thrive (i.e., bone-metastatic breast cancer and other carcinomas) owing to its rich tumor-promoting environment [2].

## 4 Traditional Therapies Available for Primary Bone Cancer

### 4.1 Chemotherapy

The cytotoxic drugs such as doxorubicin, methotrexate, cisplatin, ifosfamide, and cyclophosphamide have been at the forefront of bone cancer chemotherapy [32]. Chemotherapy is the most common pharmaceutical option for bone cancer, often regarded as first-line treatment [12]. Neoadjuvant chemotherapy and adjuvant chemotherapy are two forms of therapy given before and after surgical treatment, respectively. Neoadjuvant chemotherapy is utilized to control the mass of the primary tumor before surgical intervention; meanwhile, adjuvant chemotherapy is aimed at limiting the chances of tumor recurrence post-surgery [12,32]. The chemotherapeutic drugs used can be given individually or in combination, depending on the degree of the bone malignancy and overall patient status [33].

Essentially, combinational chemotherapy is a key component of standard multimodal therapy including surgery in patients with only primary bone tumors (without metastases), which yields a nearly 70% survival rate in comparison to patients with metastases, who have about 20% survival rate [32,33]. Nonetheless, the chemotherapy regimens still present with adverse side effects, such as neutropenia, cardiotoxicity, acute fever, as well as hypersensitivity reactions [34]. Moreover, chemotherapy-associated adverse events weigh heavily on patients, directly affecting quality of life and functionality, and interfere with the intensity of treatment protocol, leading to increased risk of tumor recurrence [34]. Accordingly, strategies to address these adverse effects include the application of drug carriers that can deliver the drugs without compromising their clinical efficacy.

### 4.2 Surgery

Surgical intervention is another available modality for the management of bone tumors [12,32]. The standard protocol for surgical intervention in bone cancer entails resection of the primary tumor, then reconstruction and ultimately adjuvant therapy depending on the type of the tumor [32]. The adjuvant therapy is included in the protocol in order to minimize the chances of tumor recurrence. Most importantly, to further reduce the risk of local recurrence when addressing malignant tumors, the surgical margins need to be wide and radical [33]. Additionally, patient-specific surgical solutions are informed by taking into account the residual estimated life expectancy [12,35]. Despite the increased local tumor control rates reported, wide resection may still result in considerable functional impairments due to the development of bone defects and discomfort in the adjacent joint [33]. As such, surgical resection on its own is not always curative and may occasionally result in tumor recurrence, hence it is generally used as a palliative intervention to relieve pain from the tumor mass [32,33].

### 4.3 Radiotherapy

Radiation therapy and radiopharmaceuticals can also be used to eliminate cancerous cells, either alone or in combination with chemotherapy and/or surgery [36]. Radiotherapy is based on the use of ionizing radiation to kill tumor cells through passing electrically charged particles. The charged particles directly kill the tumor cells by disrupting the genetic material and halting further division and growth [12,36]. Particularly, radiotherapy is used as the primary local control measure or together with surgery for the treatment of Ewing’s sarcoma [32]. Additionally, radiotherapy may be beneficial in palliative settings for improving the quality of life and skeletal function, with minimal analgesic requirements and reduced bone pain [32]. Generally, radiotherapy shows relative efficacy as adjuvant therapy in unresectable and incompletely resected tumors, for the relief of inflammation and local bone pain [36,37]. Nonetheless, although radiotherapy and radiation-enhancing materials have gained attention, the inability to control the damage of the administered radiation to healthy tissues results in undesired side effects [38].

## 5 Drug Delivery Approaches in Treating Primary Bone Cancer

### 5.1 Drug Delivery to the Bone Tissue

**Figure 3:**
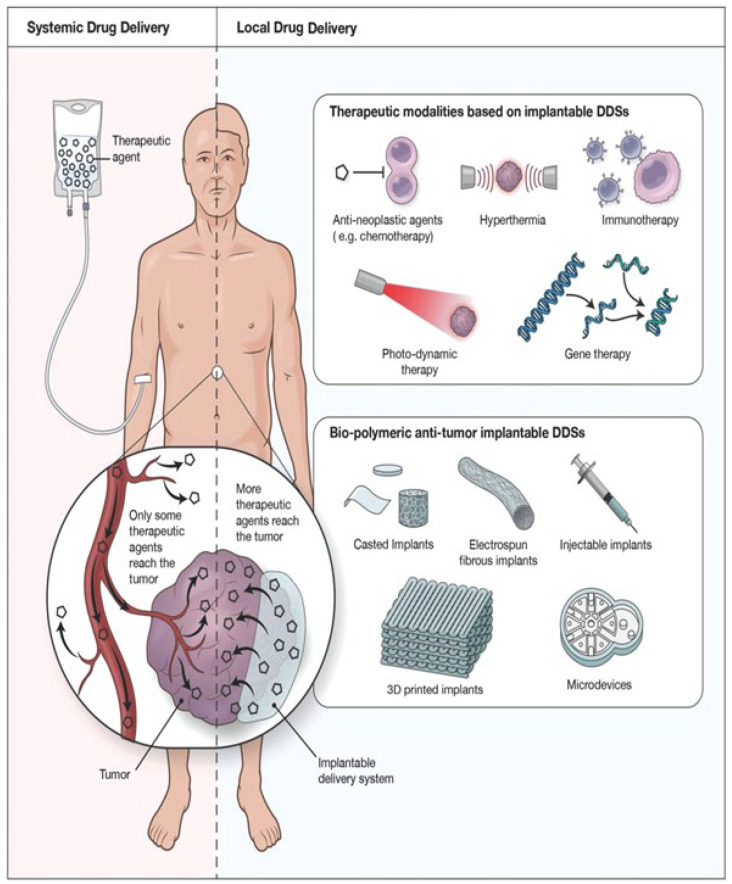
**Overview of systemic drug delivery and local drug delivery approaches for the treatment of bone tumors.** The latter employs various forms of drug-eluting implants for the execution of specific therapeutic modalities. The suitability of each approach is often dependent on the physicochemical attributes of the administered drugs and clinical circumstances, thus one may be preferable over the other. Reproduced with permission from [40]. Copyright ©2018 WILEY-VCH Verlag GmbH & Co. KGaA, Weinheim.

Anticancer drugs can be delivered to the bone tissue through a local or systemic delivery approach [15]. Local drug delivery (LDD) approach entails direct injection of drugs into the bone tissue or the application of drug-eluting implants [16,39], whereas systemic drug delivery (SDD) relates to the administration of drugs via the bloodstream [16]. Depicted in Fig. 3 is the overview of SDD and LDD as strategies used to deliver drugs against bone tumors. LDD generally involves the use of polymeric drug carriers (i.e., scaffolds, wafers, forms, and fibers), which allow for a controlled drug release to cancerous cells [39,40]. Meanwhile, SDD may involve the use of carriers that can locate cancerous cells in the bone tissue via passive (i.e., exploiting permeable tumor vasculature) or active (i.e., using targeting moieties) targeting [39].

LDD approach often presents several advantages, from the drug vehicles that are employed. Importantly, the vehicles employed in LDD are mostly biodegradable (do not require surgical removal), and are capable of achieving sustained or controlled drug release, and result in increased drug doses in tumors, with low to no adverse effects to healthy tissue [39,41]. Meanwhile, carriers used in SDD are typically based on synthetic nanomaterials such as nanoliposomes, micelles, and dendrimers, as well as cell-mediated nanoparticles for direct drug delivery [39]. These carriers can potentially facilitate the eradication of primary tumors and halt further metastases. Moreover, the carriers can locate tumors through passive or active targeting, and the release of drugs to target sites may be mediated by a specific stimulus such as temperature, pH, enzyme, light, mechanical force, or ultrasonic vibrations [39,42].

Cell-mediated drug carriers are also an excellent choice for controlled drug delivery to bone tissue, owing to their unique physiological properties and admirable biocompatibility [42]. Essentially, cell candidates may include stem cells, platelets, leukocytes, and red blood cells, with the blood constituting abundant platelets which can navigate the tumor microenvironment (TME) and can potentially detect and interact with circulating tumor cells [42,43]. Accordingly, the platelets can be conjugated with targeting moieties, such as monoclonal antibodies, to halt tumor growth and limit tumor recurrence and metastasis post surgery [43].

### 5.2 Targeted Drug Delivery Nanosystems

**Figure 4:**
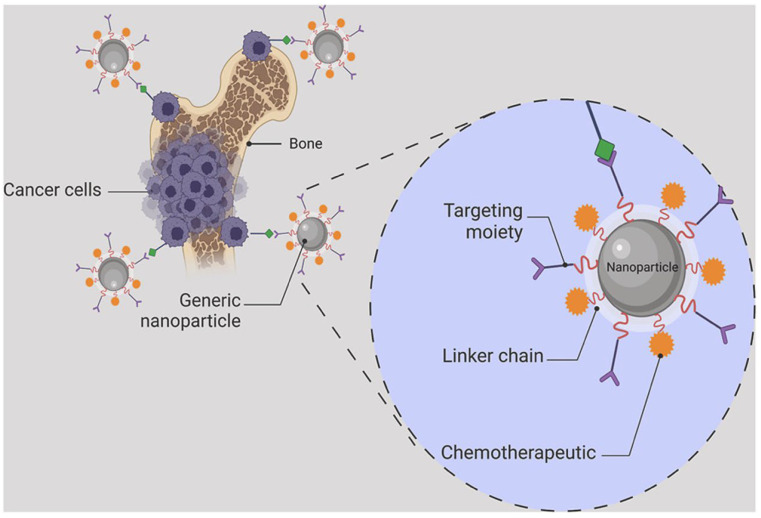
**Graphical representation of a typical nanocarrier designed for bone cancer-targeted drug delivery.** Generic nanoparticles are functionalized with a targeting moiety that specifically binds to the receptors on bone cancer cells to facilitate precise delivery of a chemotherapeutic agent. Reproduced with permission from [46]. Copyright ©2023 Elsevier B.V.

As already discussed, the clinical outcomes of the majority of anticancer drugs used in bone cancer management are compromised by non-specific and poor distribution in the tumorous cells [12,44]. Consequently, optimized drug carriers with specific targeting abilities have been devised for targeted drug delivery to bone tumors [44,45]. Nanoparticles have been exploited as drug carriers to improve the therapeutic efficacy of anticancer drugs already used in bone cancer treatment [14]. Some of the advantages presented by nanoparticles include the high loading capacity of anticancer drugs and molecules, protection of loaded drugs from biological barriers, and the ability to adequately penetrate cancerous cells and deposit drug molecules [14,46]. Additionally, the nanocarriers can be easily modified and decorated with tumor-specific ligands for active drug delivery, as depicted in Fig. 4 [46].

Many drug delivery nanocarriers designed for targeted drug delivery involve a single specific targeting moiety that binds to one specific molecular target in the TME to achieve a mono-target delivery [14,46]. For instance, surface-modified poly (D, L-lactide-co-glycolic) acid (PLGA) nanoparticles decorated with an amino-biphosphate (BP) for cabazitaxel delivery have been developed, with a higher affinity (8-fold) to bone tumors compared to the non-specific counterpart [47]. Likewise, polymeric and calcium phosphate nanoparticles, and multi-walled carbon nanotubes have been decorated with BP to target bone, and shown that the BP groups impart intrinsic bone-binding affinity to the nanocarrier [45]. Additionally, polymeric micelles loaded with anticancer drugs, and functionalized with zoledronic acid or alendronate demonstrate enhanced bone tissue accumulation [48].

Alternatively, dual target delivery can be achieved with the incorporation of ligands that can bind two receptors and mediate targeting accuracy, such as drug carriers comprising two ligands that target the bone and tumors, respectively [45]. Essentially, dual-targeting drug carriers are based on ligands that exploit the cell’s specific receptor overexpression and unique molecular structures in the TME [49,50]. One example is the overexpression of folate receptors (FR), cluster of differentiation 44 (CD44), and integrin αVβ3 in the bone cancer cells, with significant quantities and activity in tumors than in healthy cells [45,51], thus, folate (an FR target ligand) is particularly used in the modification of targeted nanocarriers in conjunction with BP for specific targeting of metastatic bone lesions [49].

Another example is the dual-targeted paclitaxel nanocarrier comprising a hydrophobic PLGA core modified with alendronate and folic acid-linked D-a-tocopheryl polyethylene glycol succinate (TPGS) coat, which showed adequate accumulation in bone tumors from IV injection, and significantly halted 4T1 cell proliferation [52]. Likewise, a dual-targeted liposomal nanocarrier for doxorubicin, modified with folate and aspartate (with high bone affinity), yielded an enhanced liposome accumulation in the tumors and bone microenvironment [53]. The inclusion of aspartate mediated the bone-targeting affinity, while folate reportedly influenced the site-specific accumulation of the liposomes in tumors. In essence, these results demonstrated that dual-target nanocarriers can be exploited for optimal specific-targeting of both bone tissue and tumors, and achieve high drug accumulation [53].

The drug delivery nanosystems can be designed and optimised to provide controlled release of cargo, triggered by different kinds of stimuli including pH, redox, light, magnetic field, and ultrasound [50,52,54]. Table 1 presents stimuli-responsive targeted drug nanocarriers that have been explored in bone cancer treatment. The well-designed chemical composition and physical properties of the nanocarriers allow selective release of the drugs in the TME, which enhances the therapeutic efficacy while minimizing unwanted side effects to normal tissues [50,54]. For example, the pH in tumors and subcellular compartments is around 5.0 and 6.0, and the use of acid-sensitive linkers (i.e., hydrazone, glycerol ester groups, acetal) can stabilize anticancer drugs at physiological pH and selectively release the drugs at acidic TME [55]. Likewise, with redox-responsive nanocarriers, glutathione (GSH) is commonly incorporated to reduce the disulfide bonds in the nanocarriers for intracellularly mediated drug release [55]. The GSH concentration in tumor cells is significantly elevated than in healthy cells, and the contrast in the redox potential is exploited to achieve selective drug release once the drug is internalized in the tumor [54,55].

**Table 1:** Stimuli-responsive targeted drug delivery nanosystems explored for the management of bone cancer.

Nanocarrier	Loaded Drug(s)	Ligand(s)	Stimulus	Ref.
Nanoliposome	doxorubicin	aspartate folate hyaluronic acid alendronate	redox	[53,56]
Mesoporous silica NPs	doxorubicin	zoledronic acid	pH	[57,58]
Calcium phosphate NPs	methotrexate	alendronate	/	[59]
Polymeric NPs	cabazitaxel doxorubicin paclitaxel	zoledronic acid alendronate hyaluronic acid folate	redox pH	[47,52,55,60]
Micelles	docetaxel bortezomib	alendronate quinolone nonpeptide	pH	[61,62]
Dendrimers	docetaxel bortezomib	alendronate tripeptide Arg-Gly-Asp (RGD) hyaluronic acid	redox pH	[63,64]
Nano-metal organic frameworks	zoledronate	folate	pH	[49]

NPs: nanoparticles.

## 6 Innovative Nano-Based Drug Delivery Systems

### 6.1 Current Marketed Therapy

The therapeutic benefits of several marketed chemotherapeutics employed in the treatment of bone cancer have long been known to be compromised by poor tumor accumulation and distribution, owing to non-specificity and rapid systemic clearance [45]. Since most chemotherapy protocols involve systemic drug delivery (drugs administered through the bloodstream), it becomes crucial for the administered drugs to be able to navigate through the systemic barriers while in circulation [45,65]. Notably, drug carriers like nanoparticles are capable of shielding the drugs from biological barriers and achieving site-specific delivery by locating cancerous cells via active or passive targeting [66]. Several nano-based drug carriers, such as dendrimers, micelles, liposomes, and polymeric nanoparticles, have since been developed for application in other cancers [12], however, only one nano-based drug has been approved by the European Medicines Agency (EMA) and is currently marketed for application in bone cancer treatment.

**Figure 5:**
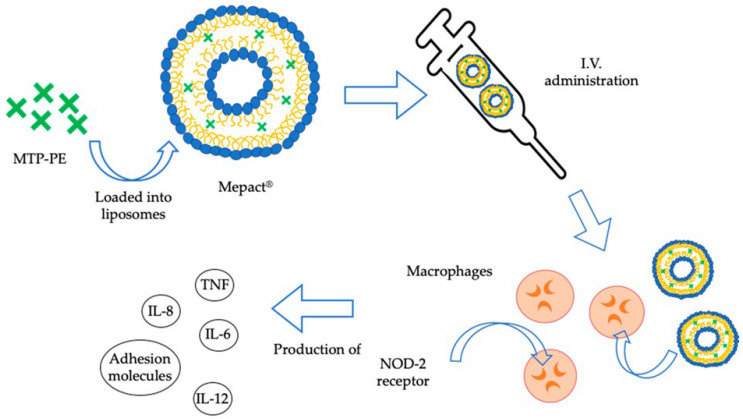
**Simplistic mechanism of action of MEPACT^®^ for the treatment of osteosarcoma.** The nanomedicine works by activating macrophages through binding to the nucleotide-binding oligomerization domain-containing protein 2 (NOD2), leading to cytokine (i.e., interleukins and tumor-necrosis factor) and tumoricidal activity, which helps eliminate residual osteosarcoma cells when used alongside chemotherapy. Adapted with permission from [69], under the Creative Commons Attribution (CC BY) license. Copyright ©2020 MDPI, Basel, Switzerland.

Mifamurtide, a liposomal muramyl tripeptide phosphatidyl ethanolamine (MEPACT^®^) remains the only nano-based drug currently approved (EMA) for the treatment of bone cancer [67]. Muramyl tripeptide phosphatidyl ethanolamine (MTP-PE) is a lipophilic derivative of muramyl dipeptide, which, due to its lipophilic properties, can be encapsulated into liposomes, yielding an effective antitumor nanodrug [68]. Accordingly, the liposomal formulation of MTP-PE, mifamurtide (MEPACT^®^), manufactured by Takeda Pharmaceutical Company Limited, Japan, was approved in 2009 for use in young adults and children for the treatment of osteosarcoma, following surgical resection [67]. The formulation is administered along with postoperative multi-agent chemotherapy. A simplified mechanism of action is depicted in Fig. 5. Although the drug is not endorsed by the FDA, osteosarcoma patients in the United States of America can still obtain MEPACT^®^ through the FDA’s compassionate use and personal importation programmes [68].

### 6.2 Promising Innovative Drug Nanocarriers

**Figure 6:**
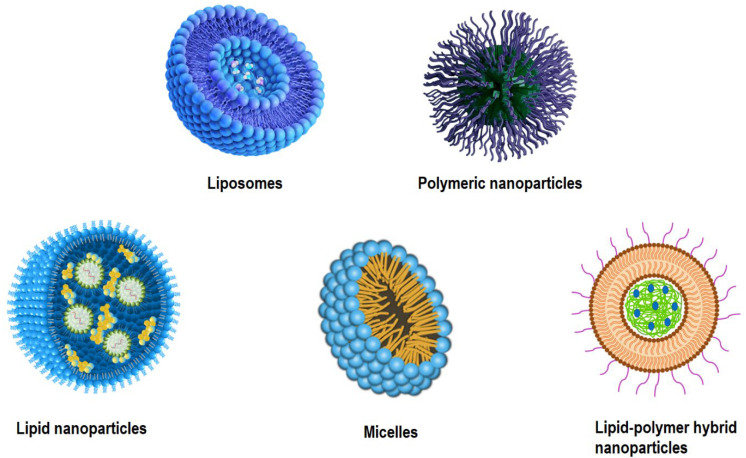
**Graphical depiction of the model drug nanocarriers that have been investigated for optimised drug delivery for the treatment of primary bone cancer.** These nanocarriers present with high drug loading capacity, inherent biocompatibility, and physical stability, amongst the key attributes that make them desirable for drug delivery.

The Nanoscale drug delivery systems are currently being investigated for the optimization of bone cancer treatment [11]. Several nanomaterials that possess desirable physicochemical properties, such as biocompatibility, high drug loading capacity, and stability, have been used to develop a variety of drug nanocarriers with potential application in bone cancer treatment [11,45]. Amongst the innovative drug nanocarriers that have been investigated for bone cancer treatment are liposomes, lipid-based nanoparticles, polymeric micelles, and lipid-polymer hybrid nanoparticles. These are graphically depicted in Fig. 6 and further discussed below. Selected examples of promising innovative drug nanocarriers for the treatment of bone cancer are presented in Table 2. The designed nanocarriers are ideal for drug delivery in solid tumors due to the small size (i.e., 100–300 nm), allowing passive accumulation into tumors [11].

**Table 2:** Promising innovative drug nanocarriers for the treatment of bone cancer.

Nanocarrier	Particle Size	Drug	Significance	*In Vivo* Data	*In Vitro* Data	Ref.
Liposomes	173.1 nm	Doxorubicin	Enhanced tumor penetration and targeted delivery of doxorubicin	Antitumor efficacy; Histological evaluation	Cytotoxicity (MTT assay); Cellular uptake	[56]
Polymeric nanoparticles (i.e., polyethylene glycol, PEG)	160 nm	Doxorubicin	Greater inhibition of tumor growth *in vivo*, when compared to free drug.	Antitumor activity; Targeting ability	Cell cycle progression; Apoptosis	[60]
Lipid nanoparticles	136 nm	Ifosfamide	Nanoparticles exhibited a narrow size distribution, high drug loading capacity	N/A	Cytotoxicity (MTT assay); Subcellular localization	[70]
Micelles	28 nm	Doxorubicin	High anti-proliferative activity in human osteosarcoma cell line (Saos-2)	N/A	Cytotoxicity	[71]
Lipid-polymer hybrid nanoparticles	100 nm	Doxorubicin	Superior antitumor efficacy compared to single drug	Antitumor efficacy; Histological analysis	Cytotoxicity; Cellular uptake	[72]

MTT: (3-(4, 5-dimethylthiazol-2-yl)-2,5-diphenyltetrazolium bromide); N/A: not available; PEG: polyethylene glycol.

#### 6.2.1 Liposomes

Liposomes have been consistently favoured over the years for anticancer drug delivery, owing to their biocompatibility, biodegradability, and non-toxicity [73]. These spherical vesicles present with flexibility to load both hydrophobic and hydrophilic anticancer drugs, as they are made up of phospholipids and cholesterol, forming one or more phospholipid bilayers, allowing both hydrophilic and hydrophobic drug loading [74]. The number of phospholipid bilayers as well as vesicle size greatly influence the half-life of liposomal drug formulations and drug encapsulation efficiency [73,74]. A hybrid liposomal formulation for the delivery of doxorubicin was reported by Federman et al. [75], which exhibited specific targeting to osteosarcoma. The formulation incorporated the osteosarcoma-associated cell surface antigen (ALCAM) as a targeting ligand, forming an anti-ALCAM hybrid system with enhanced cytotoxic activity compared to non-targeted doxorubicin hybrid liposomes [75]. Another promising osteosarcoma-targeted liposomal system was developed by Feng et al. [56], functionalized with a cluster of differentiation 44 (CD44)-targeting moiety for the direct delivery of doxorubicin. The targeted liposomal system exhibited enhanced accumulation at the osteosarcoma site with increased overall survival *in vivo* [56].

#### 6.2.2 Polymeric Nanoparticles

Polymeric nanoparticles comprising biodegradable polymers such as chitosan, polyethylene glycol (PEG), and poly(lactic-co-glycolic acid) (PLGA), have attracted notable attention as promising drug nanocarriers for bone cancer treatment [76]. These polymeric nanocarriers can be used for both dual or single delivery of anticancer drugs, and can be surface modified with various ligands that target overexpressed receptors on cancerous cells for site-specific drug delivery [76]. Chitosan nanoparticles loaded with a DNA enzyme (Dz13) showed promising outcomes in two distinct osteosarcoma animal models, resulting in tumor growth regression, non-toxicity, and reduced osteolysis [77]. Likewise, PLGA-alendronate (ALN) nanoparticles loaded with doxorubicin have been developed, and reportedly show more efficacy in preventing osteolytic bone metastasis, compared to the free drug [78]. Similarly, novel doxorubicin-conjugated biphosphnate nanoparticles comprising PEG have been designed for the treatment of primary bone tumors, and demonstrated enhanced tumor targetability and antitumor efficacy in a xenograft mouse model [60].

#### 6.2.3 Lipid Nanoparticles

Lipid nanoparticles represent a significant class of promising drug nanocarriers for cancer management, including bone cancer [73]. This class, consisting of solid lipid nanoparticles (SLNs), core-shell lipid nanoparticles (CLNs), lipid nanocapsules (LNCs), and nanoemulsions, shows promise in promoting anticancer drug bioavailability, regulating drug release and delivery to target areas, and providing enhanced intracellular permeability [73,79]. Primarily, lipid nanoparticles present with significant advantages for application as anticancer drug carriers, including biodegradability, biocompatibility, and colloidal stability [79]. Accordingly, Wang et al. (2018) reported on the formulation of ifosfamide-encapsulated LNCs for the treatment of osteosarcoma. These demonstrated an enhanced cytotoxicity in MG63 osteosarcoma cells [70].

#### 6.2.4 Micelles

One of the innovative and promising drug nanocarriers in cancer treatment is polymeric micelles, derived from co-polymers with hydrophobic and hydrophilic units that form structural complexes with a hydrophobic core and a stabilizing hydrophilic shell [76]. Polymeric micelles are commonly exploited in anticancer drug delivery for their characteristic extravasation from blood vessels and tumor accumulation due to small size [19]. Accordingly, several drug-loaded polymeric micelles have been investigated for anticancer treatment [19,71,76]. A promising micellar drug nanocarrier was developed by Low and co-workers for the delivery of doxorubicin in human osteosarcoma cells [71]. The nanocarrier comprised the hydrophobic core of 11-aminoundecanoic acid and the hydrophilic shell of D-aspartic acid octapeptide, linked with 8-amino-3, 6-dioxaoctanoic acid. It was reported that the developed micellar doxorubicin nanocarrier demonstrated promising cytotoxic activity and tumor targeting efficacy [71].

#### 6.2.5 Lipid-Polymer Hybrid Nanoparticles

Lipid-polymer hybrid nanoparticles (LPHNPs) are innovative nanocarriers that combine the structural advantages of both lipid and polymer-based nanoparticles, to yield a “hybrid” with the capabilities to circumvent the drawbacks of these nano-formulations [80,81]. As such, the exploitation of LPHNPs as alternative nanocarriers in bone cancer is gaining huge attention owing to their inherent biocompatibility, stability, prolonged circulation time, controlled drug release, and favourable *in vivo* efficacy [81]. LPHNPs are able to efficiently load both hydrophilic and hydrophobic compounds, and allow for controlled drug release. The lipid component is favourable for enhanced drug loading and membrane permeability; meanwhile, the polymer plays a key role in mediating the drug release [80]. In 2015, Ramasamy and co-workers designed a lipid-polymer hybrid complex for the co-delivery of doxorubicin and irinotecan, and reported that the complex exhibited significant antitumor activity compared to the single drugs [72].

## 7 Challenges in Clinical Translation of Nanocarriers for Bone Cancer Treatment

Despite the remarkable progress in nanomedicine over the years, the clinical translation of anticancer drug nanocarriers remains limited, in contrast to the huge number of preclinical studies conducted [82]. Although several nanocarriers, such as liposomes, polymeric nanoparticles, lipid nanoparticles, micelles, and lipid-polymer hybrid nanoparticles, have demonstrated therapeutic promise in bone cancer [73,76,80], only a few have reached the market. A notable example includes a liposomal formulation of MTP-PE, mifamurtide (MEPACT^®^), approved for the treatment of osteosarcoma [67]. Fundamentally, the gap between bench and bedside arises from a myriad of translational challenges, including large-scale production, physicochemical stability, immunogenicity, and long-term toxicity [82,83,84]. This section discusses these challenges and aptly highlights potential strategies to address them.

### 7.1 Large-Scale Production

Nanocarriers are mostly developed using laboratory-scale techniques such as nanoprecipitation, emulsification, and thin-film hydration, which can be difficult to scale without altering critical quality attributes (CQAs) like particle size, polydispersity index (PDI), surface charge, encapsulation efficiency, and drug release kinetics [84,85]. Moreover, minor variations in mixing rate, temperature, solvent evaporation, or polymer molecular weight can significantly impact nanoparticle characteristics [85]. Particularly, liposomes may exhibit size heterogeneity during scale-up due to shear-dependent lipid bilayer formation; meanwhile, polymeric nanoparticles may show altered drug loading due to solvent diffusion kinetics [82,86]. Moving forward, these challenges may be potentially addressed through the adoption of Quality by Design (QbD) frameworks and application of scalable and continuous manufacturing techniques, such as continuous flow reactors, high-pressure homogenization, and microfluidics-based systems [82,83].

### 7.2 Stability

The challenge of instability with most nanocarriers is a multifaceted issue encompassing physical, chemical, and biological aspects [83,87]. Common physical instability issues include aggregation or fusion (which mostly affects liposomes and polymeric nanoparticles) and premature drug leakage (which affects lipid nanoparticles and micelles) [87]. These issues may arise upon dilution in physiological fluids and during storage or transportation. On the other hand, chemical degradation of lipids and polymers through oxidation and hydrolysis, respectively, are common chemical instability issue that may compromise the therapeutic efficacy of nanocarriers [83,84]. The biological instability of nanocarriers, often observed from their interaction with plasma proteins when administered systemically, alters their cellular uptake and biodistribution, and ultimately reduces therapeutic efficacy [83,88]. Essentially, the use of surface modification methodologies, such as PEGylation and incorporation of antioxidants in lipid systems, may be explored for addressing the stability challenge that most nanocarriers face [89].

### 7.3 Immunogenicity and Immunotoxicity

In essence, nanocarriers can activate the immune system in unintended ways [83,90]. Certain nanocarriers (i.e., lipid-based, polymeric systems) may trigger complement activation-related pseudoallergy (CARPA), leading to infusion reactions [90]. It is reported that PEGylated nanoparticles, though designed to evade immune recognition, can induce anti-PEG antibodies upon repeated administration [91]. Meanwhile, inorganic nanocarriers and cationic polymers may activate inflammatory pathways, including NOD-like receptor pyrin domain-containing protein 3 (NLRP3) inflammasome signaling [90]. Importantly, surface charge plays a major role, with highly cationic nanoparticles often more immunostimulatory [90]. As a solution to unintended innate and adaptive immune responses, rigorous screening for complement activation during preclinical development and incorporation of immunomodulatory components when appropriate may be explored [83,90].

### 7.4 Long-Term Toxicity and Biodistribution

Non-biodegradable nanomaterials such as gold, quantum dots, and certain silica particles may accumulate in the liver, spleen, or lymph nodes, raising concerns about chronic toxicity [82,83]. Likewise, even biodegradable systems may generate potentially toxic degradation products (e.g., PLGA degrades into lactic and glycolic acid, which may cause local acidosis in high concentrations) [92]. In the long run, these may result in fibrosis or granuloma formation, persistent oxidative stress, as well as interfere with normal organ function [82]. To address this, designing biodegradable nanocarriers with predictable degradation pathways may be explored, and comprehensive metabolic profiling and dose optimization to prevent the accumulation of degradation products should be implemented.

## 8 Conclusion and Future Prospects

Despite constituting less than 1% of the global cancer cases, the aggressive progression of primary bone cancer poses a concerning medical challenge. Currently, the effective treatment of bone cancer is challenging, mainly compromised by the complexity of bone tissue and inefficient delivery and biodistribution of anticancer drugs into tumors. Moreover, chemotherapy and surgical interventions are constantly associated with significant challenges. Particularly, chemotherapy as the first-line treatment option presents with major disadvantages, such as the looming drug resistance by cancer cells, as well as non-specificity, resulting in poor pharmacokinetics and a myriad of side effects. As such, the advent of drug nanocarriers for the systemic delivery of anticancer drugs presents a promising avenue towards the effective treatment of bone tumors. Accordingly, several innovative drug delivery nanosystems, such as liposomes, polymeric nanoparticles, micelles, lipid nanoparticles, and lipid-polymer hybrid nanocomplexes, show positive clinical outcomes in pre-clinical and clinical trials, for possible application in bone cancer treatment. Although there is only one liposomal-based therapy (MEPACT^®^) currently marketed for bone cancer treatment, it is envisaged that more innovative therapies will emerge soon, based on the promising clinical data.

The development of targeted and efficient drug delivery systems remains a critical focus in advancing bone cancer treatment. Given the limitations of conventional therapies, the continued exploration of nanocarrier-based drug delivery systems offers a promising avenue for overcoming challenges such as poor bioavailability, drug resistance, and off-target toxicity. Future research should focus on optimizing nanocarrier formulations, improving tumor-targeting capabilities, and enhancing drug release mechanisms to maximize therapeutic efficacy. Additionally, the integration of personalized medicine approaches, including biomarker-driven therapies and gene-editing techniques, holds great potential in tailoring treatments to individual patients for improved therapeutic outcomes.

## Data Availability

Not applicable.

## References

[1] Lai J , Li X , Liu W , Qian L , Zhong C . Global, regional, and national burden and trends analysis of malignant neoplasm of bone and articular cartilage from 1990 to 2021: A systematic analysis for the global burden of disease study 2021. Bone. 2024; 188: 117212. doi:10.1016/j.bone.2024.117212. 39059750

[2] Hosseini H , Heydari S , Hushmandi K , Daneshi S , Raesi R . Bone tumors: A systematic review of prevalence, risk determinants, and survival patterns. BMC Cancer. 2025; 25( 1): 321. doi:10.1186/s12885-025-13720-0. 39984867 PMC11846205

[3] Al Sawaftah NM , Pitt WG , Husseini GA . Incorporating nanoparticles in 3D printed scaffolds for bone cancer therapy. Bioprinting. 2023; 36: e00322. doi:10.1016/j.bprint.2023.e00322.

[4] Long N , Woodlock D , D’Agostino R , Nguyen G , Gangai N , Sevilimedu V , et al. Incidence and prevalence of bone metastases in different solid tumors determined by natural language processing of CT reports. Cancers. 2025; 17( 2): 218. doi:10.3390/cancers17020218. 39858000 PMC11763382

[5] Zhang X , Dai X , Chen Y , Wang S , Yang H , Qu B , et al. Global, regional, and national burden of malignant neoplasm of bone and articular cartilage in adults aged 65 years and older, 1990–2021: A systematic analysis based on the global burden of disease study 2021. Aging Clin Exp Res. 2025; 37( 1): 21. doi:10.1007/s40520-024-02926-0. 39776003 PMC11711276

[6] Olajugba OJ , Oladeji EO , Adesola D , Abdullateef RO , Rockson G , Bah AK , et al. Challenges of osteosarcoma care in Africa: A scoping review of the burden, management and outcome. Ecancermedicalscience. 2025; 19: 1835. doi:10.3332/ecancer.2025.1835. 40177151 PMC11959130

[7] Siegel RL , Miller KD , Wagle NS , Jemal A . Cancer statistics, 2023. CA Cancer J Clin. 2023; 73( 1): 17– 48. doi:10.3322/caac.21763. 36633525

[8] Lindsey BA , Markel JE , Kleinerman ES . Osteosarcoma overview. Rheumatol Ther. 2017; 4( 1): 25– 43. doi:10.1007/s40744-016-0050-2. 27933467 PMC5443719

[9] Longhi A , Benassi MS , Molendini L , Macchiagodena M , Picci P , Bacci G . Osteosarcoma in blood relatives. Oncol Rep. 2001; 8( 1): 131– 6. doi:10.3892/or.8.1.131. 11115584

[10] Sadykova LR , Ntekim AI , Muyangwa-Semenova M , Rutland CS , Jeyapalan JN , Blatt N , et al. Epidemiology and risk factors of osteosarcoma. Cancer Investig. 2020; 38( 5): 259– 69. doi:10.1080/07357907.2020.1768401. 32400205

[11] Ambrosio L , Raucci MG , Vadalà G , Ambrosio L , Papalia R , Denaro V . Innovative biomaterials for the treatment of bone cancer. Int J Mol Sci. 2021; 22( 15): 8214. doi:10.3390/ijms22158214. 34360979 PMC8347125

[12] Bădilă AE , Rădulescu DM , Niculescu AG , Grumezescu AM , Rădulescu M , Rădulescu AR . Recent advances in the treatment of bone metastases and primary bone tumors: An up-to-date review. Cancers. 2021; 13( 16): 4229. doi:10.3390/cancers13164229. 34439383 PMC8392383

[13] Durfee RA , Mohammed M , Luu HH . Review of osteosarcoma and current management. Rheumatol Ther. 2016; 3( 2): 221– 43. doi:10.1007/s40744-016-0046-y. 27761754 PMC5127970

[14] Giordano F , Lenna S , Rampado R , Brozovich A , Hirase T , Tognon MG , et al. Nanodelivery systems face challenges and limitations in bone diseases management. Adv Ther. 2021; 4( 12): 2100152. doi:10.1002/adtp.202100152.

[15] Anupama Devi VK , Ray S , Arora U , Mitra S , Sionkowska A , Jaiswal AK . Dual drug delivery platforms for bone tissue engineering. Front Bioeng Biotechnol. 2022; 10: 969843. doi:10.3389/fbioe.2022.969843. 36172012 PMC9511792

[16] Aquib M , Juthi AZ , Farooq MA , Ali MG , Janabi AHW , Bavi S , et al. Advances in local and systemic drug delivery systems for post-surgical cancer treatment. J Mater Chem B. 2020; 8( 37): 8507– 18. doi:10.1039/D0TB00987C. 32839803

[17] Ngema LM , Adeyemi SA , Choonara YE . 3-Bone cancer: Current and new drug delivery systems. In: Shegokar R , Pathak YV , editors. Drug delivery landscape in cancer research. Cambridge, MA, USA: Academic Press; 2025. p. 53– 69. doi:10.1016/B978-0-443-29168-5.00019-2.

[18] Šromová V , Sobola D , Kaspar P . A brief review of bone cell function and importance. Cells. 2023; 12( 21): 2576. doi:10.3390/cells12212576. 37947654 PMC10648520

[19] Amiryaghoubi N , Fathi M , Barar J , Omidian H , Omidi Y . Advanced nanoscale drug delivery systems for bone cancer therapy. Biochim Biophys Acta Mol Basis Dis. 2023; 1869( 6): 166739. doi:10.1016/j.bbadis.2023.166739. 37146918

[20] Xu H , Zhao Q , Miao X , Zhu L , Wang J . Clinical decision-making in bone cancer care management and forecast of ICU needs based on computed tomography. J Bone Oncol. 2024; 49: 100646. doi:10.1016/j.jbo.2024.100646. 39559513 PMC11570866

[21] Kim C , Davis LE , Albert CM , Samuels B , Roberts JL , Wagner MJ . Osteosarcoma in pediatric and adult populations: Are adults just big kids? Cancers. 2023; 15( 20): 5044. doi:10.3390/cancers15205044. 37894411 PMC10604996

[22] Rathore R , Van Tine BA . Pathogenesis and current treatment of osteosarcoma: Perspectives for future therapies. J Clin Med. 2021; 10( 6): 1182. doi:10.3390/jcm10061182. 33809018 PMC8000603

[23] Crombé A , Simonetti M , Longhi A , Hauger O , Fadli D , Spinnato P . Imaging of osteosarcoma: Presenting findings, metastatic patterns, and features related to prognosis. J Clin Med. 2024; 13( 19): 5710. doi:10.3390/jcm13195710. 39407770 PMC11477067

[24] Kim JH , Lee SK . Classification of chondrosarcoma: From characteristic to challenging imaging findings. Cancers. 2023; 15( 6): 1703. doi:10.3390/cancers15061703. 36980590 PMC10046282

[25] Gazendam A , Popovic S , Parasu N , Ghert M . Chondrosarcoma: A clinical review. J Clin Med. 2023; 12( 7): 2506. doi:10.3390/jcm12072506. 37048590 PMC10095313

[26] Zöllner SK , Amatruda JF , Bauer S , Collaud S , de Álava E , DuBois SG , et al. Ewing sarcoma-diagnosis, treatment, clinical challenges and future perspectives. J Clin Med. 2021; 10( 8): 1685. doi:10.3390/jcm10081685. 33919988 PMC8071040

[27] Mishra MN , Sharma R , Chandavarkar V , Premalatha BR . Pathogenesis of Ewing sarcoma: Existing and emerging trends. Adv Cancer Biol Metastasis. 2021; 2: 100008. doi:10.1016/j.adcanc.2021.100008.

[28] Zare F , Shahbazi N , Faraji N , Goli R , Mostafaei B , Anari S . A cruel invasion of Ewing’s sarcoma of the skull: A rare case report. Int J Surg Case Rep. 2023; 108: 108380. doi:10.1016/j.ijscr.2023.108380. 37406533 PMC10382727

[29] Florencio-Silva R , da Silva Sasso GR , Sasso-Cerri E , Simões MJ , Cerri PS . Biology of bone tissue: Structure, function, and factors that influence bone cells. Biomed Res Int. 2015; 2015( 1): 421746. doi:10.1155/2015/421746. 26247020 PMC4515490

[30] Tam WL , Luyten FP , Roberts SJ . From skeletal development to the creation of pluripotent stem cell-derived bone-forming progenitors. Philos Trans R Soc Lond B Biol Sci. 2018; 373( 1750): 20170218. doi:10.1098/rstb.2017.0218. 29786553 PMC5974441

[31] Zhu S , Chen W , Masson A , Li YP . Cell signaling and transcriptional regulation of osteoblast lineage commitment, differentiation, bone formation, and homeostasis. Cell Discov. 2024; 10: 71. doi:10.1038/s41421-024-00689-6. 38956429 PMC11219878

[32] Rajani R , Gibbs CP . Treatment of bone tumors. Surg Pathol Clin. 2012; 5( 1): 301– 18. doi:10.1016/j.path.2011.07.015. 22328909 PMC3273870

[33] Bickels J , Campanacci DA . Local adjuvant substances following curettage of bone tumors. J Bone Joint Surg Am. 2020; 102( 2): 164– 74. doi:10.2106/JBJS.19.00470. 31613863

[34] Lustberg MB , Kuderer NM , Desai A , Bergerot C , Lyman GH . Mitigating long-term and delayed adverse events associated with cancer treatment: Implications for survivorship. Nat Rev Clin Oncol. 2023; 20( 8): 527– 42. doi:10.1038/s41571-023-00776-9. 37231127 PMC10211308

[35] Sambri A , Caldari E , Fiore M , Zucchini R , Giannini C , Pirini MG , et al. Margin assessment in soft tissue sarcomas: Review of the literature. Cancers. 2021; 13( 7): 1687. doi:10.3390/cancers13071687. 33918457 PMC8038240

[36] Baskar R , Lee KA , Yeo R , Yeoh KW . Cancer and radiation therapy: Current advances and future directions. Int J Med Sci. 2012; 9( 3): 193– 9. doi:10.7150/ijms.3635. 22408567 PMC3298009

[37] De Felice F , Piccioli A , Musio D , Tombolini V . The role of radiation therapy in bone metastases management. Oncotarget. 2017; 8( 15): 25691– 9. doi:10.18632/oncotarget.14823. 28148890 PMC5421962

[38] Zhang S , Wang X , Gao X , Chen X , Li L , Li G , et al. Radiopharmaceuticals and their applications in medicine. Signal Transduct Target Ther. 2025; 10( 1): 1. doi:10.1038/s41392-024-02041-6. 39747850 PMC11697352

[39] Kortam S , Lu Z , Zreiqat H . Recent advances in drug delivery systems for osteosarcoma therapy and bone regeneration. Commun Mater. 2024; 5: 168. doi:10.1038/s43246-024-00612-2.

[40] Talebian S , Foroughi J , Wade SJ , Vine KL , Dolatshahi-Pirouz A , Mehrali M , et al. Biopolymers for antitumor implantable drug delivery systems: Recent advances and future outlook. Adv Mater. 2018; 30( 31): e1706665. doi:10.1002/adma.201706665. 29756237

[41] Ding L , Agrawal P , Singh SK , Chhonker YS , Sun J , Murry DJ . Polymer-based drug delivery systems for cancer therapeutics. Polymers. 2024; 16( 6): 843. doi:10.3390/polym16060843. 38543448 PMC10974363

[42] Chen Y , Wu X , Li J , Jiang Y , Xu K , Su J . Bone-targeted nanoparticle drug delivery system: An emerging strategy for bone-related disease. Front Pharmacol. 2022; 13: 909408. doi:10.3389/fphar.2022.909408. 35712701 PMC9195145

[43] Yu H , Yang Z , Li F , Xu L , Sun Y . Cell-mediated targeting drugs delivery systems. Drug Deliv. 2020; 27( 1): 1425– 37. doi:10.1080/10717544.2020.1831103. 33096949 PMC7594730

[44] Wang Y , Wang C , Xia M , Tian Z , Zhou J , Berger JM , et al. Engineering small-molecule and protein drugs for targeting bone tumors. Mol Ther. 2024; 32( 5): 1219– 37. doi:10.1016/j.ymthe.2024.03.001. 38449313 PMC11081876

[45] Cheng X , Wei J , Ge Q , Xing D , Zhou X , Qian Y , et al. The optimized drug delivery systems of treating cancer bone metastatic osteolysis with nanomaterials. Drug Deliv. 2021; 28( 1): 37– 53. doi:10.1080/10717544.2020.1856225. 33336610 PMC7751395

[46] Ashique S , Faiyazuddin M , Afzal O , Gowri S , Hussain A , Mishra N , et al. Advanced nanoparticles, the hallmark of targeted drug delivery for osteosarcoma—An updated review. J Drug Deliv Sci Technol. 2023; 87: 104753. doi:10.1016/j.jddst.2023.104753.

[47] Gdowski AS , Ranjan A , Sarker MR , Vishwanatha JK . Bone-targeted cabazitaxel nanoparticles for metastatic prostate cancer skeletal lesions and pain. Nanomedicine. 2017; 12( 17): 2083– 95. doi:10.2217/nnm-2017-0190. 28805551 PMC5585843

[48] Hatami E , Bhusetty Nagesh PK , Chowdhury P , Elliot S , Shields D , Chand Chauhan S , et al. Development of zoledronic acid-based nanoassemblies for bone-targeted anticancer therapy. ACS Biomater Sci Eng. 2019; 5( 5): 2343– 54. doi:10.1021/acsbiomaterials.9b00362. 33405784

[49] Au KM , Satterlee A , Min Y , Tian X , Kim YS , Caster JM , et al. Folate-targeted pH-responsive calcium zoledronate nanoscale metal-organic frameworks: Turning a bone antiresorptive agent into an anticancer therapeutic. Biomaterials. 2016; 82: 178– 93. doi:10.1016/j.biomaterials.2015.12.018. 26763733 PMC4728024

[50] Yan S , Na J , Liu X , Wu P . Different targeting ligands-mediated drug delivery systems for tumor therapy. Pharmaceutics. 2024; 16( 2): 248. doi:10.3390/pharmaceutics16020248. 38399302 PMC10893104

[51] Assaraf YG , Leamon CP , Reddy JA . The folate receptor as a rational therapeutic target for personalized cancer treatment. Drug Resist Updat. 2014; 17( 4–6): 89– 95. doi:10.1016/j.drup.2014.10.002. 25457975

[52] Chen SH , Liu TI , Chuang CL , Chen HH , Chiang WH , Chiu HC . Alendronate/folic acid-decorated polymeric nanoparticles for hierarchically targetable chemotherapy against bone metastatic breast cancer. J Mater Chem B. 2020; 8( 17): 3789– 800. doi:10.1039/D0TB00046A. 32150202

[53] Ke X , Lin W , Li X , Wang H , Xiao X , Guo Z . Synergistic dual-modified liposome improves targeting and therapeutic efficacy of bone metastasis from breast cancer. Drug Deliv. 2017; 24( 1): 1680– 9. doi:10.1080/10717544.2017.1396384. 29092646 PMC8241154

[54] Wang SY , Hu HZ , Qing XC , Zhang ZC , Shao ZW . Recent advances of drug delivery nanocarriers in osteosarcoma treatment. J Cancer. 2020; 11( 1): 69– 82. doi:10.7150/jca.36588. 31892974 PMC6930408

[55] Zhao YP , Ye WL , Liu DZ , Cui H , Cheng Y , Liu M , et al. Redox and pH dual sensitive bone targeting nanoparticles to treat breast cancer bone metastases and inhibit bone resorption. Nanoscale. 2017; 9( 19): 6264– 77. doi:10.1039/C7NR00962C. 28470315

[56] Feng S , Wu ZX , Zhao Z , Liu J , Sun K , Guo C , et al. Engineering of bone- and CD44-dual-targeting redox-sensitive liposomes for the treatment of orthotopic osteosarcoma. ACS Appl Mater Interfaces. 2019; 11( 7): 7357– 68. doi:10.1021/acsami.8b18820. 30682240

[57] Sun W , Han Y , Li Z , Ge K , Zhang J . Bone-targeted mesoporous silica nanocarrier anchored by zoledronate for cancer bone metastasis. Langmuir. 2016; 32( 36): 9237– 44. doi:10.1021/acs.langmuir.6b02228. 27531422

[58] Qiao H , Cui Z , Yang S , Ji D , Wang Y , Yang Y , et al. Targeting osteocytes to attenuate early breast cancer bone metastasis by theranostic upconversion nanoparticles with responsive plumbagin release. ACS Nano. 2017; 11( 7): 7259– 73. doi:10.1021/acsnano.7b03197. 28692257

[59] Chu W , Huang Y , Yang C , Liao Y , Zhang X , Yan M , et al. Calcium phosphate nanoparticles functionalized with alendronate-conjugated polyethylene glycol (PEG) for the treatment of bone metastasis. Int J Pharm. 2017; 516( 1–2): 352– 63. doi:10.1016/j.ijpharm.2016.11.051. 27887884

[60] Rudnick-Glick S , Corem-Salkmon E , Grinberg I , Margel S . Targeted drug delivery of near IR fluorescent doxorubicin-conjugated poly(ethylene glycol) bisphosphonate nanoparticles for diagnosis and therapy of primary and metastatic bone cancer in a mouse model. J Nano Biotechnol. 2016; 14( 1): 80. doi:10.1186/s12951-016-0233-6. PMC5139040 27919267

[61] Zhu J , Huo Q , Xu M , Yang F , Li Y , Shi H , et al. Bortezomib-catechol conjugated prodrug micelles: Combining bone targeting and aryl boronate-based pH-responsive drug release for cancer bone-metastasis therapy. Nanoscale. 2018; 10( 38): 18387– 97. doi:10.1039/C8NR03899F. 30256367

[62] Ross MH , Esser AK , Fox GC , Schmieder AH , Yang X , Hu G , et al. Bone-induced expression of integrin β3 enables targeted nanotherapy of breast cancer metastases. Cancer Res. 2017; 77( 22): 6299– 312. doi:10.1158/0008-5472.CAN-17-1225. 28855208 PMC5841166

[63] Wang M , Cai X , Yang J , Wang C , Tong L , Xiao J , et al. A targeted and pH-responsive bortezomib nanomedicine in the treatment of metastatic bone tumors. ACS Appl Mater Interfaces. 2018; 10( 48): 41003– 11. doi:10.1021/acsami.8b07527. 30403331

[64] Bai SB , Liu DZ , Cheng Y , Cui H , Liu M , Cui MX , et al. Osteoclasts and tumor cells dual targeting nanoparticle to treat bone metastases of lung cancer. Nanomedicine. 2019; 21: 102054. doi:10.1016/j.nano.2019.102054. 31310809

[65] Stapleton M , Sawamoto K , Alméciga-Díaz CJ , MacKenzie WG , Mason RW , Orii T , et al. Development of bone targeting drugs. Int J Mol Sci. 2017; 18( 7): 1345. doi:10.3390/ijms18071345. 28644392 PMC5535838

[66] Bhatia S , Tykodi SS , Lee SM , Thompson JA . Systemic therapy of metastatic melanoma: On the road to cure. Oncology. 2015; 29( 2): 126– 35. 25683834

[67] Frampton JE . Mifamurtide. Pediatr Drugs. 2010; 12( 3): 141– 53. doi:10.2165/11204910-000000000-00000. 20481644

[68] Meyers PA , Chou AJ . Muramyl tripeptide-phosphatidyl ethanolamine encapsulated in liposomes (L-MTP-PE) in the treatment of osteosarcoma. In: Current advances in osteosarcoma. Cham, Switzerland: Springer International Publishing; 2014. p. 307– 21. doi:10.1007/978-3-319-04843-7_17. 24924182

[69] Chindamo G , Sapino S , Peira E , Chirio D , Gonzalez MC , Gallarate M . Bone diseases: Current approach and future perspectives in drug delivery systems for bone targeted therapeutics. Nanomaterials. 2020; 10( 5): 875. doi:10.3390/nano10050875. 32370009 PMC7279399

[70] Wang SQ , Zhang Q , Sun C , Liu GY . Ifosfamide-loaded lipid-core-nanocapsules to increase the anticancer efficacy in MG63 osteosarcoma cells. Saudi J Biol Sci. 2018; 25( 6): 1140– 5. doi:10.1016/j.sjbs.2016.12.001. 30174514 PMC6116780

[71] Low SA , Yang J , Kopeček J . Bone-targeted acid-sensitive doxorubicin conjugate micelles as potential osteosarcoma therapeutics. Bioconjug Chem. 2014; 25( 11): 2012– 20. doi:10.1021/bc500392x. 25291150 PMC4240342

[72] Ramasamy T , Ruttala HB , Choi JY , Tran TH , Kim JH , Ku SK , et al. Engineering of a lipid-polymer nanoarchitectural platform for highly effective combination therapy of doxorubicin and irinotecan. Chem Commun. 2015; 51( 26): 5758– 61. doi:10.1039/C5CC00482A. 25720556

[73] Burdușel AC , Andronescu E . Lipid nanoparticles and liposomes for bone diseases treatment. Biomedicines. 2022; 10( 12): 3158. doi:10.3390/biomedicines10123158. 36551914 PMC9775639

[74] Akbarzadeh A , Rezaei-Sadabady R , Davaran S , Joo SW , Zarghami N , Hanifehpour Y , et al. Liposome: Classification, preparation, and applications. Nanoscale Res Lett. 2013; 8( 1): 102. doi:10.1186/1556-276X-8-102. 23432972 PMC3599573

[75] Federman N , Chan J , Nagy JO , Landaw EM , McCabe K , Wu AM , et al. Enhanced growth inhibition of osteosarcoma by cytotoxic polymerized liposomal nanoparticles targeting the alcam cell surface receptor. Sarcoma. 2012; 2012( 1): 126906. doi:10.1155/2012/126906. 23024593 PMC3447386

[76] Prasad SR , Sampath Kumar TS , Jayakrishnan A . Nanocarrier-based drug delivery systems for bone cancer therapy: A review. Biomed Mater. 2021; 16( 4): 044107. doi:10.1088/1748-605X/abf7d5. 33853043

[77] Tan ML , Dunstan DE , Friedhuber AM , Choong PFM , Dass CR . A nanoparticulate system that enhances the efficacy of the tumoricide Dz13 when administered proximal to the lesion site. J Control Release. 2010; 144( 2): 196– 202. doi:10.1016/j.jconrel.2010.01.011. 20079783

[78] Salerno M , Cenni E , Fotia C , Avnet S , Granchi D , Castelli F , et al. Bone-targeted doxorubicin-loaded nanoparticles as a tool for the treatment of skeletal metastases. Curr Cancer Drug Targets. 2010; 10( 7): 649– 59. doi:10.2174/156800910793605767. 20578992

[79] Sguizzato M , Esposito E , Cortesi R . Lipid-based nanosystems as a tool to overcome skin barrier. Int J Mol Sci. 2021; 22( 15): 8319. doi:10.3390/ijms22158319. 34361084 PMC8348303

[80] Parveen S , Gupta P , Kumar S , Banerjee M . Lipid polymer hybrid nanoparticles as potent vehicles for drug delivery in cancer therapeutics. Med Drug Discov. 2023; 20: 100165. doi:10.1016/j.medidd.2023.100165.

[81] Mohanty A , Uthaman S , Park IK . Utilization of polymer-lipid hybrid nanoparticles for targeted anti-cancer therapy. Molecules. 2020; 25( 19): 4377. doi:10.3390/molecules25194377. 32977707 PMC7582728

[82] Liu Y , Zhang Y , Li H , Hu TY . Recent advances in the bench-to-bedside translation of cancer nanomedicines. Acta Pharm Sin B. 2025; 15( 1): 97– 122. doi:10.1016/j.apsb.2024.12.007. 40041906 PMC11873642

[83] Ewii UE , Attama AA , Olorunsola EO , Onugwu AL , Nwakpa FU , Anyiam C , et al. Nanoparticles for drug delivery: Insight into *in vitro* and *in vivo* drug release from nanomedicines. Nano TransMed. 2025; 4: 100083. doi:10.1016/j.ntm.2025.100083.

[84] Khalid-Salako F , Salimi Khaligh S , Fathi F , Demirci OC , Öncer N , Kurt H , et al. The nanocarrier landscape—Evaluating key drug delivery vehicles and their capabilities: A translational perspective. ACS Appl Mater Interfaces. 2025; 17( 26): 37383– 403. doi:10.1021/acsami.5c07366. 40526827 PMC12232268

[85] Wang F , Harker A , Edirisinghe M , Parhizkar M . Micro- and nanomanufacturing for biomedical applications and nanomedicine: A perspective. Small Sci. 2023; 3( 11): 2300039. doi:10.1002/smsc.202300039. 40213525 PMC11935837

[86] Desai N , Rana D , Patel M , Bajwa N , Prasad R , Vora LK . Nanoparticle therapeutics in clinical perspective: Classification, marketed products, and regulatory landscape. Small. 2025; 21( 29): 2502315. doi:10.1002/smll.202502315. 40454890 PMC12288819

[87] Mahajan K , Bhattacharya S . The advancement and obstacles in improving the stability of nanocarriers for precision drug delivery in the field of nanomedicine. Curr Top Med Chem. 2024; 24( 8): 686– 721. doi:10.2174/0115680266287101240214071718. 38409730

[88] Voke E , Arral M , Squire HJ , Lin TJ , Zheng L , Coreas R , et al. Protein corona formed on lipid nanoparticles compromises delivery efficiency of mRNA cargo. Nat Commun. 2025; 16( 1): 8699. doi:10.1038/s41467-025-63726-2. 41027853 PMC12485112

[89] Zaky MF , Hammady TM , Gad S , Alattar A , Alshaman R , Hegazy A , et al. Influence of surface-modification via PEGylation or chitosanization of lipidic nanocarriers on *in vivo* pharmacokinetic/pharmacodynamic profiles of apixaban. Pharmaceutics. 2023; 15( 6): 1668. doi:10.3390/pharmaceutics15061668. 37376116 PMC10302406

[90] La-Beck NM , Islam MR , Markiewski MM . Nanoparticle-induced complement activation: Implications for cancer nanomedicine. Front Immunol. 2021; 11: 603039. doi:10.3389/fimmu.2020.603039. 33488603 PMC7819852

[91] Kozma GT , Mészáros T , Vashegyi I , Fülöp T , Örfi E , Dézsi L , et al. Pseudo-anaphylaxis to polyethylene glycol (PEG)-coated liposomes: Roles of anti-PEG IgM and complement activation in a porcine model of human infusion reactions. ACS Nano. 2019; 13( 8): 9315– 24. doi:10.1021/acsnano.9b03942. 31348638

[92] Makadia HK , Siegel SJ . Poly lactic-co-glycolic acid (PLGA) as biodegradable controlled drug delivery carrier. Polymers. 2011; 3( 3): 1377– 97. doi:10.3390/polym3031377. 22577513 PMC3347861

